# Mutations in NA That Induced Low pH-Stability and Enhanced the Replication of Pandemic (H1N1) 2009 Influenza A Virus at an Early Stage of the Pandemic

**DOI:** 10.1371/journal.pone.0064439

**Published:** 2013-05-16

**Authors:** Tadanobu Takahashi, Jiasheng Song, Takashi Suzuki, Yoshihiro Kawaoka

**Affiliations:** 1 Influenza Research Institute, Department of Pathobiological Sciences, School of Veterinary Medicine, University of Wisconsin, Madison, Wisconsin, United States of America; 2 Department of Biochemistry, School of Pharmaceutical Sciences, University of Shizuoka, and Global COE Program for Innovation in Human Health Sciences, Shizuoka, Japan; 3 Division of Virology, Department of Microbiology and Immunology, Institute of Medical Science, University of Tokyo, Tokyo, Japan; 4 ERATO Infection-Induced Host Responses Project, Japan Science and Technology Agency, Saitama, Japan; National Institute for Viral Disease Control and Prevention, CDC, China

## Abstract

An influenza A virus that originated in pigs caused a pandemic in 2009. The sialidase activity of the neuraminidase (NA) of previous pandemic influenza A viruses are stable at low pH (≤5). Here, we identified the amino acids responsible for this property. We found differences in low-pH stability at pH 5.0 among pandemic (H1N1) 2009 viruses, which enhanced the replication of these viruses. Low-pH-stable NA enhancement of virus replication may have contributed to the rapid worldwide spread and adaptation to humans of pandemic (H1N1) 2009 viruses during the early stages of the 2009 pandemic.

## Introduction

In the spring of 2009, the pandemic (H1N1) 2009 virus emerged in Mexico and spread rapidly among humans worldwide [1]. Subtypes of influenza A virus are determined by antigenicities of the two envelope glycoproteins, hemagulutinin (HA) and neuraminidase (NA). HA binds to terminal sialic acid of glyco-conjugates on the host cell surface as a viral receptor. NA is known to facilitate progeny virus release from the host cell surface through sialidase activity, which cleaves sialic acid from glyco-conjugates. Worldwide spread of new subtype influenza A virus in humans is called “pandemic”. There were three pandemics in the 20^th^ century: H1N1 Spanish flu in 1918, H2N2 Asian flu in 1957, and H3N2 Hong Kong flu in 1968. Influenza A virus has eight-segmented RNA genomes called PB2, PB1, PA, HA, nucleoprotein (NP), NA, M, and NS. New subtype viruses, which are candidates of pandemic virus, are thought to occur by reassortment of segmented RNA genomes between human virus and other host virus in an intermediate host such as pigs. Multiple factors are associated with the emergence of pandemic influenza viruses including their replicative ability in humans and their antigenicity. For pandemic (H1N1) 2009 virus, the role of mutations in PB2, PB1-F2 (a frame-shift product of PB1 gene), PA, HA, NP, and NS1 has been shown in virus replicability and pathogenicity in cell culture and animals [2], [3]; however, the properties of the NA of pandemic (H1N1) 2009 virus are largely unknown with the exception of its resistance to the sialidase inhibitors zanamivir and oseltamivir, which inhibit progeny virus release from the host cell surface.

We previously showed that influenza virus NAs differ in their stability at low pH (≤5). All avian virus NAs tested to date are highly stable at low pH; their sialidase activities are retained even after pre-incubation for 10 min at pH 5.0 or less [4]. The NAs of pandemic human viruses, such as 1918 H1N1, 1957 H2N2, and 1968 H3N2 viruses, are also low-pH-stable. On the other hand, the NAs of most seasonal human influenza A viruses (IAVs) are unstable at low pH [4]–[7]. Viruses possessing a low-pH-stable NA from a pandemic IAV in the background of A/WSN/1933 (WSN; H1N1) replicated more efficiently in cell culture and mouse lungs compared with a WSN virus possessing a low-pH-unstable NA [8]. Furthermore, we found that the NA of the 1968 pandemic H3N2 virus was low-pH-stable, and that this property disappeared from human H3N2 viruses after 1971 [6]. This research also suggested that a low-pH-stable NA might contribute to a pandemic and play an important role in the adaptation of human viruses.

Here, we examined the low-pH stability of the sialidase activity of the pandemic (H1N1) 2009 viruses. We found differences in the pH stability among their NAs. We also identified the amino acid determinants that confer low-pH stability to pandemic (H1N1) 2009 viruses and used a reverse genetics approach to show that low-pH-stable NA enhances virus replication.

## Materials and Methods

### Cells

Human embryonic kidney 293T cells were maintained in high glucose Dulbecco’s modified medium supplemented with 10% fetal bovine serum (FBS). Madin-Darby canine kidney (MDCK) cells were maintained in Eagle’s minimum essential medium supplemented with 5% FBS. Human lung adenocarcinoma Calu-3 cells (kindly provided by Raymond Pickles, University of North Carolina) were maintained in a 1:1 mixture of Dulbecco’s modified medium and Ham’s F12 nutrient medium (DF12; Invitrogen, Carlsbad, CA) supplemented with 10% FBS.

### NA genes and plasmids

Pandemic (H1N1) 2009 virus, A/California/04/2009 (Cal04), A/Wisconsin/WSLH26327/2009 (WisWSLH), A/Norway/3568/2009 (Nor3568), and A/Norway/3858/2009 (Nor3858) were cloned from virus by extracting viral RNA and performing reverse transcription-PCR with primers specific for the NA genes. The NA genes were inserted into the multicloning region between the *Eco*R I site and the *Xho* I site of the expression plasmid pCAGGS/MCS vector [9], between the two *BsmB* I sites of the expression plasmid pCAGGS/BsmBI vector [10], or between the two *BsmB* I sites of the plasmid pHH21 vector [9]. The V106I and N248D mutations of Cal04 NA were introduced by means of PCR. All NA genes were sequencing using specific primers.

### Sialidase activity of cell-expressed NA

293T cells (1.5×10^5^ cells/well) in a 24-well tissue culture plate were cultured overnight. The following day, the 70% confluent cells were transfected with a plasmid (1 µg/well) for NA expression by using TransIT-293 (Mirus, Madison, WI). After a 24-h incubation at 37°C, the transfected cells were suspended in phosphate-buffered saline (PBS; 1.2 ml/well), and 50 µl of each cell suspension was transferred into microtubes and centrifuged at 100 ×*g* for 10 min. The cell pellets were incubated with 57 µl of 10 mM acetate buffer (pH 4.0, 5.0, or 6.0) at 37°C for 10 min. Here, when we measured pH profiles of sialidase activities, the transfected cells at 24 h were suspended in PBS (1.2 ml/well), and 15 µl of each cell suspension was transferred into microtubes and centrifuged at 100 ×*g* for 10 min. The cell pellets were incubated with 57 µl of 10 mM acetate buffer (pH 4.0, 4.5, 5.0, 5.5, or 6.0) or 10 mM phosphate buffer (pH 6.0, 6.5, 7.0, 7.5, or 8.0) was used instead of 57 µl of 10 mM acetate buffer (pH 4.0, 5.0, or 6.0). Fifty microliters of each suspension was then transferred to a 96-well black plate on ice and reacted with 2.5 µl of 2 mM 2′-(4-methylumbelliferyl)-*N*-acetylneuraminic acid (Sigma-Aldrich Corp., St. Louis, MI) at 37°C for 30 min. The reaction was stopped by the addition of 200 µl of 100 mM sodium carbonate buffer (pH 10.7). The fluorescence intensity (Ex, 355 nm; Em, 460 nm) was measured with an Infinite M1000 microplate reader (Tecan Group Ltd., Männedorf, Switzerland). The sialidase activities of the cell-expressed NA were expressed as a percentage of the activity at pH 6.0.

### Generation of reverse genetics viruses

Reverse genetics was performed in the backbone of Cal04 by using the pHH21 vector containing the wild-type NA gene or a mutated Cal04NA gene (2 mutations: from Val to Ile at position 106 and from Asn to Asp at position 248 based on Cal04 NA numbering), together with seven plasmids (pHH21-PB2, PB1, PA, HA, NP, M, and NS) from Cal04. The viruses were propagated using MDCK cells in serum-free medium (SFM), Hybridoma-SFM (Invitrogen Corp., Carlsbad, CA) containing TPCK-trypsin (1 µg/ml). The NA genes of the virus stocks obtained were sequencing by using specific primers.

For growth curves, MCDK cells (1×10^5^ cells/well) or Calu-3 cells (1×10^5^ cells/well) in a 24-well plate were cultured overnight. The cells were then infected with viruses at a multiplicity of infection of 0.005 (plaque forming unit/cell) for 30 min at 37°C. After being washed with PBS, the MDCK cells and the Calu-3 cells were cultured in SFM containing TPCK-trypsin (1 µg/ml) and a 1∶1 mixture of Dulbecco’s modified medium and Ham’s F12 nutrient medium containing TPCK-trypsin (0.5 ug/ml) and 0.3% bovine serum albumin, respectively. The virus titers in the supernatant at 17, 26, 43, and 52 h post-infection were measured by means of plaque assays.

### Plaque assay

MDCK cells (2.0×10^5^ cells/well) in a 6-well plate were cultured overnight. The confluent cell monolayers were then washed and incubated for 30 min at 37°C with log dilutions of virus in SFM. The infected monolayers were then overlaid with a solution of SFM containing TPCK-trypsin (1 μg/ml) and 0.5% agarose. The monolayers were incubated at 37°C for 2 days and then fixed with 2 ml/well of 10% formalin solution at room temperature overnight. To visualize viral plaques, the fixed cells were incubated with 1% Crystal Violet solution in 20% methanol and then washed with water. To visualize viral foci, the viral antigens in the infected cells were reacted with a rabbit anti-A/WSN/1933 (H1N1) polyclonal antibody (R309) for 30 min at room temperature and then with horseradish peroxidase-conjugated goat anti-rabbit IgG (Invitrogen Corp., Carlsbad, CA) for 30 min at room temperature. The infected cells were stained by using 3,3'-diaminobenzidine, tetrahydrochloride. Each plaque was measured by using Image J release 1.40 g (National Institutes of Health, USA, http://rsb.info.nih.gov/ij/) from scan image.

### Phylogenic tree

The phylogenic tree was generated from the NA open reading frame nucleotide sequences of 65 pandemic (H1N1) 2009 viruses and 15 human H1N1 viruses isolated in the 2010/2011 season, together with four swine influenza A viruses used as an out-group to differentiate between the early and late stages in the evolution of pandemic (H1N1) 2009 NA (Table S1), by using DNASTAR Lasergene software (DNASTAR, Inc. Madison, WI).

## Results

### Low-pH stabilities of the sialidase activities of pandemic (H1N1) 2009 virus NAs

**Figure 1:**
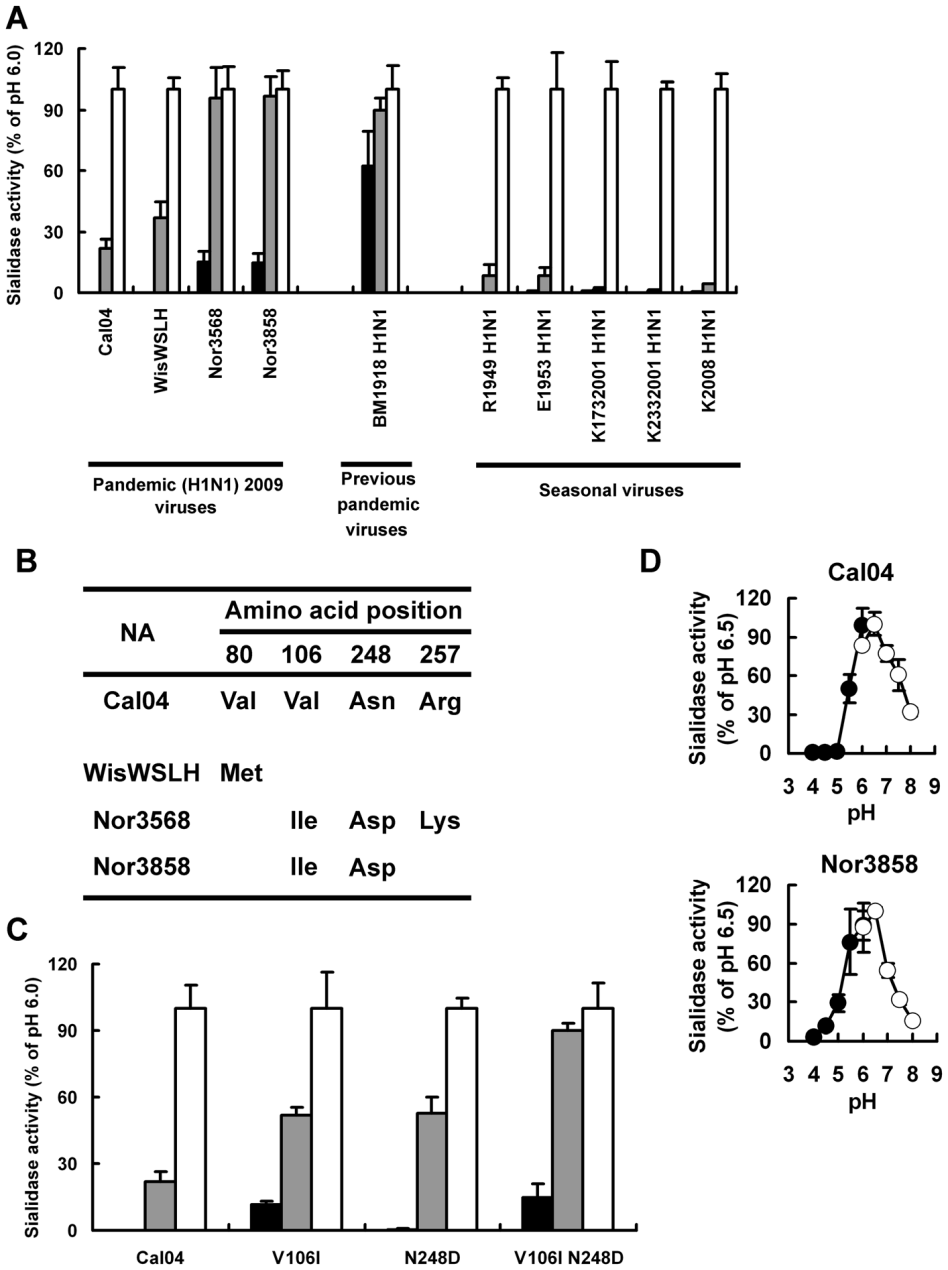
The low-pH stabilities of pandemic (H1N1) 2009 virus NAs and Cal04 NA mutants. A, Low-pH stabilities of NA-expressing cells transfected with a plasmid expressing NA from pandemic (H1N1) 2009 viruses exposed to pH 4.0 (closed column), 5.0 (hatched column) and 6.0 (open column). Sialidase activities are expressed as a percentage of each activity at pH 6.0. Results with BM1918 H1N1 NA and seasonal H1N1 NAs are cited from reference [11]. B, Amino acid comparison of the pandemic (H1N1) 2009 virus NAs with Cal04 NA. Residues different from Cal04 NA are indicated for each NA except Cal04 NA (Cal04 N1 numbering). C, Low-pH stabilities of NA-expressing cells transfected with Cal04 NA mutants. Sialidase activities (%) are expressed as described in (A). D, pH profiles of sialidase activities of Cal04 NA and Nor3858 NA. Ten mM acetate buffer (closed circle) and 10 mM phosphate buffer (open circle) were used for pH 4.0–6.0 and pH 6.0–8.0, respectively. Sialidase activities (%) are expressed as a percentage of that at pH 6.5, which is the highest activity within pH 4.0–8.0.

All of the NAs of the avian viruses tested and the pandemic viruses of 1918, 1957, and 1968 were low-pH-stable [4]–[8]. Since the pandemic (H1N1) 2009 virus NA originated from a Eurasian avian-like swine virus, which was introduced into European pigs from a bird in 1979 [1], the pandemic (H1N1) 2009 virus NA may have retained its low-pH stability. To test this possibility, we examined the low-pH stabilities of the sialidase activities of pandemic (H1N1) 2009 virus NAs by using cell-expressed NA after pre-incubation at pH 4.0, 5.0, or 6.0. None of the NAs of the four pandemic (H1N1) 2009 viruses we tested showed the same low-pH stability of the previous pandemic virus A/Brevig Mission/1/1918 (H1N1) (BM1918 H1N1) [7]. However, the low-pH stabilities of these pandemic (H1N1) 2009 virus NAs appeared to be somewhat higher than those of seasonal H1N1 NAs [A/Rome/1949 (R1949 H1N1), A/England/1953 (E1953 H1N1), A/Kawasaki/173/2001 (K1732001 H1N1), A/Kawasaki/233/2001 (K2332001 H1N1), and A/Kawasaki/23/2008 (K2008 H1N1)], which lost sialidase activity after exposure to pH 4.0 and 5.0 [7], [11]; the NAs of Nor3568 and Nor3858 however retained high sialidase activity at pH 5.0 and even showed activity at pH 4.0. On the other hand, the NAs of Cal04 and WisWSLH had reduced activity at pH 5.0 and lost activity at pH 4.0 (Figure 1A). The decrease in sialidase activity at pH 4.0 and 5.0 may be due to an optimum pH shift toward a high pH. We therefore checked pH profiles of the sialdiase activities of Cal04 NA and Nor3858 NA. Both NAs showed optimum pH within the range of pH 6.0–6.5 (Figure 1D). The low-pH stability did not result from an optimum pH shift. This result coincided with results of our previous study showing that the low-pH stability in N2 NA did not result from an optimum pH shift [4].

### Identification of the amino acid residues responsible for the low-pH stability of pandemic (H1N1) 2009 virus NA

A comparison of the NA amino acid sequences revealed that the NAs of pandemic (H1N1) 2009 viruses had different residues at positions 80, 106, 248, and 257 (Figure 1B). Since there were two amino acid differences between the low-pH-unstable NA of Cal04 and the low-pH-stable NA of Nor3858 at pH 5.0, the Ile at position 106 and the Asp at position 248 of Nor3858 NA were candidate determinants for the acquisition of low-pH stability. To identify the amino acid residues responsible for the low-pH stability of some of the pandemic (H1N1) 2009 virus NAs, we generated three mutants of Cal04 NA: one with a single mutation at position 106 from Val to Ile (V106I), one with a single mutation at position 248 from Asn to Asp (N248D), and one with two of these mutations. We then measured the low-pH stabilities of the mutant NAs (Figure 1C). The low-pH stability of the Cal04 NA mutant with both V106I and N248D was similar to that of Nor3568 NA and Nor3858 NA, indicating that both the V106I and the N248D mutations were responsible for the low-pH stability of pandemic (H1N1) 2009 virus NA.

### Comparison of the replicability of reverse genetics-generated pandemic (H1N1) 2009 viruses possessing low-pH-stable NA or low-pH-unstable NA

**Figure 2:**
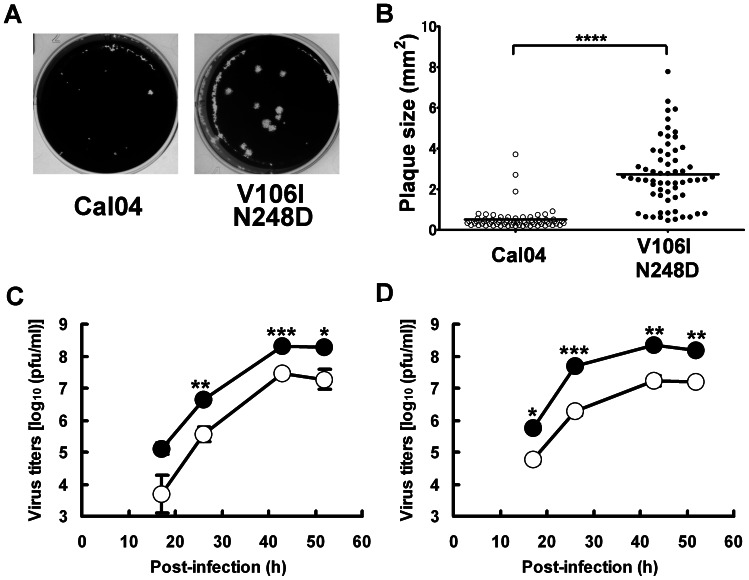
Comparison of plaque size and virus replication between the wild-type and an NA mutant virus. Cal04 denotes the wild-type virus and V106I N248D denotes the NA mutant virus with both the V106I and N248D of Cal04. A, Images of plaque formation. B, Comparison of plaque size. Cal04, n = 67; V106I N248D, n = 62. C, Growth curves in MDCK cells. D, Growth curves in Calu-3 cells. Closed circles, V106I N248D; open circles, Cal04. The Mann-Whitney U test and paired *t*-test were used for statistical analysis in (B) and (C and D), respectively. *. *P*<0.05; **, *P*<0.01; ***, *P*<0.001; ****, *P*<0.0001.

We previously demonstrated that the low-pH-stable NA of human influenza A virus of subtypes N1 and N2 can enhance virus replication [5], [7]. To test whether the low-pH stability of pandemic (H1N1) 2009 virus NA also enhances virus replication, we produced two pandemic (H1N1) 2009 viruses possessing wild-type Cal04 NA or mutated Cal04 NA with the V106I and N248D mutations by using reverse genetics in the backbone of Cal04. The wild-type Cal04 formed very small plaques, whereas the Cal04 NA mutant with V106I and N248D formed clear large plaques (Figure 2A). Viral antigens in these plaques were confirmed by immunostaining (data not shown). There was a statistically significant difference in plaque size between the two viruses (Figure 2B). The Cal04 NA mutant showed approximately 10 times higher virus titers than did the wild-type at each time point of the growth curve, not only in MDCK cells (Figure 2C), but also in Calu-3 cells (Figure 2D), which were reported to show high replicability of pandemic (H1N1) 2009 virus [12]. These results indicate that the low-pH stability of pandemic (H1N1) 2009 virus NA appears to enhance virus replication, and that pandemic (H1N1) 2009 viruses circulating in 2009 possessed NAs of different low-pH stabilities that are directly linked to virus replicability.

## Discussion

**Figure 3:**
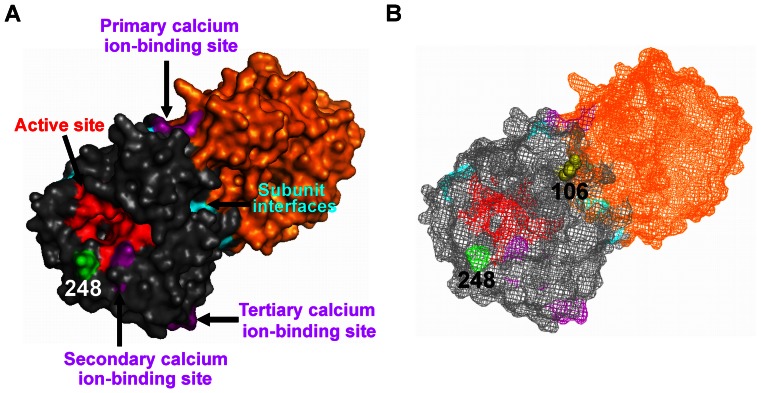
Identification of amino acids responsible for the low-pH stability of pandemic (H1N1) 2009 virus NA. A, A single subunit of the NA homodimer structure (3NSS. pdb, Cal04) is shown in gray and orange. In the surface models of NA, red, purple, and cyan indicate the active site, the calcium ion-binding site, and the subunit interfaces, respectively. The residue at position 248 is colored green (Cal04 N1 numbering). B, Structure of the NA homodimer shown in (A) presented as a mesh model. The residue at position 106 is shown as a yellow sphere (Cal04 N1 numbering). Pictures were generated by using the Pymol Molecular Graphics System Ver. 1.1r1 (DeLano Scientific LLC).

Monomeric Cal04 NA contains an active site (positions 118, 119, 151, 152, 179, 180, 223, 225, 275, 277, 278, 293, 295, 345, 368, and 402), subunit interfaces (positions 99, 104, 139, 205, 215, 454, 458, and 459) and three calcium-ion binding sites (the primary site at positions 111 and 113; the secondary site at positions 294, 298, 324, 342, and 344; and the tertiary site at positions 319, 376, 378, 384, and 386) [13]–[15]. Position 248, a surface residue, is located near to both the active site and the secondary calcium ion-binding site (Figure 3A), which is thought to be involved in the conformation of the active site [16], [17]. Position 106, an inner residue, is located near to both the primary calcium ion-binding site and the subunit interfaces (Figure 3B). We previously identified the residues at positions 430, 435, and 454 (BM1918 H1N1 N1 numbering) as important for the low-pH stability of N1 NAs [7] and the residues at positions 344 and 466 (1968 H3N2 N2 numbering) as important for the low-pH stability of N2 NAs [5]; these residues are also located near the enzymatic active site, the calcium ion-binding site, and the subunit interfaces. These findings suggest that similar mechanisms exist by which influenza virus NAs become stable at low pH that are subtype-independent, although the specific amino acid residues necessary for this conversion may differ among the subtypes.

**Figure 4:**
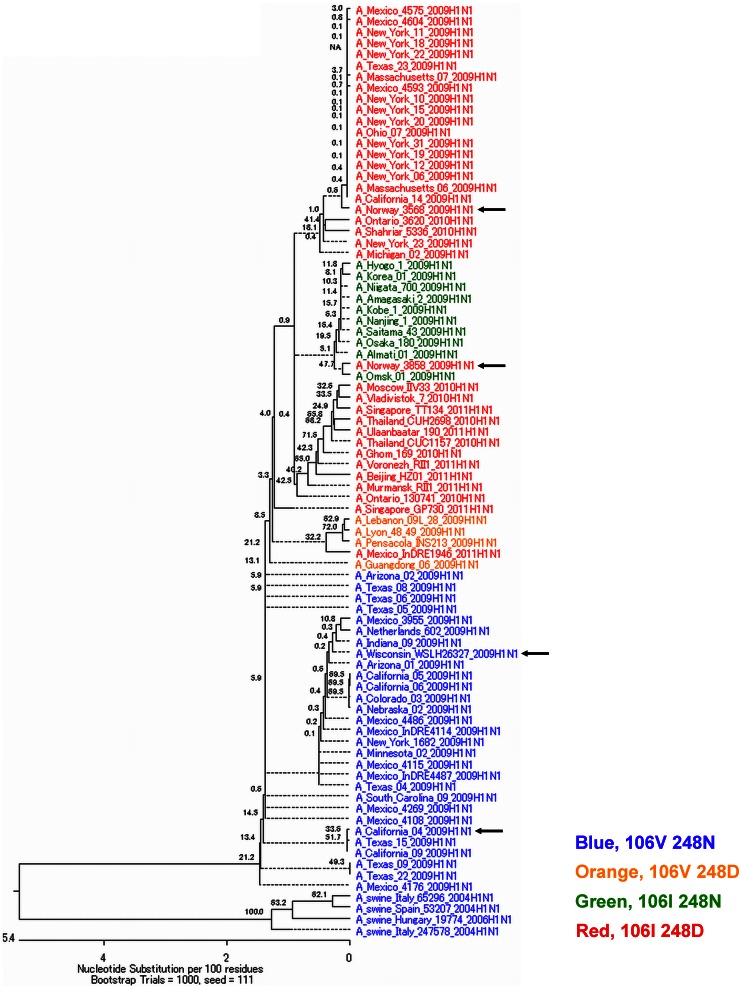
Phylogenic tree of the NA open reading frame nucleotide sequences. Pandemic (H1N1) 2009 viruses and human H1N1 viruses isolated in the 2010/2011 season were analyzed. Arrows indicate the pandemic (H1N1) 2009 viruses used in this study. Viruses are colored-coded according to the amino acid residues at positions 106 and 248; Blue, 106V and 248N; Orange, 106V and 248D; Green, 106I and 248N; and Red, 106I and 248D.

To determine how the low-pH-stable pandemic (H1N1) 2009 virus NAs emerged during the evolution of this virus, we looked for the V106I and N248D mutations in a phylogenic analysis of pandemic (H1N1) 2009 virus NAs. Low-pH-stable NA with Ile and Asp at positions 106 and 248, respectively, appears to have evolved from low-pH-unstable NA with Val and Asn at these positions in several different lineages (Figure 4). Viruses with Ile and Asp at positions 106 and 248, respectively, in NA were isolated between 19 April, 2009 and 12 March, 2011, whereas viruses with Val and Asn at these positions in NA were isolated between 30 March, 2009 and 29 April, 2009, with some isolated in early April 2009 (Table S1). This suggests that pandemic (H1N1) 2009 viruses with both the V106I and N248D mutations in NA, many of which were isolated from late April 2009 in the United States (Table S1), must have arisen rapidly after infections in humans with the pandemic (H1N1) 2009 virus that originated around March 2009. Given that the low-pH stability of NA enhances virus replication, the acquisition of low-pH-stable NA might have contributed to the rapid worldwide spread and adaptation to humans of pandemic (H1N1) 2009 virus during the early stage of the 2009 pandemic. Interestingly, our phylogenetic analysis revealed two intermediates carrying either the V106I or the N248D mutation in NA. Both intermediates ultimately acquired both mutations (Figure 4), which were apparent in isolates from September 2010 to March 2011 (89 of 90 NAs in GenBank, Table S2). This finding suggests that acquisition of both the V106I and N248D mutation allows for efficient adaptation of the pandemic (H1N1) 2009 virus to humans. However, the low-pH stability of pandemic (H1N1) 2009 virus NA may be lost within several years in order for the virus to adapt to create a long-term epidemic in humans, as has been observed with previous pandemics [6].

For the reverse genetic virus possessing N2 NA, at pH 6.0, absolute sialidase activity of the low-pH-stable NA of 1968 H3N2 virus was approximately two-times lower than that of the low-pH-unstable NA with two mutations of arginine to lysine at 344 and of phenylalanine to leucine at 466 (1968 H3N2 N2 numbering) of the corresponding virus. At pH 6.0, absolute sialidase activity of the low-pH-unstable NA of 1968 H2N2 virus was similar to that of the low-pH-stable NA with a mutation of leucine to phenylalanine at 466 of the corresponding virus. Of these viruses, the viruses with low-pH-stable NA showed high replicability in MDCK cells and in a mouse model compared to the viruses with low-pH-unstable NA [8]. In the present study, when the same amounts of NA genes were transfected, fluorescent intensities resulting from sialidase activity at pH 6.0 were 16272 (±1680) for Cal04 NA and 13859 (±1558) for Cal04 V106 N248D NA. The results of a paired *t*-test showed that there was no significant difference between these activities. Taken together, the results suggest that enhancement of virus replicability by low-pH-stable NA is not associated with absolute sialidase activity.

Pandemic (H1N1) 2009 virus with low-pH-stable NA formed much larger plaques than did the virus with low-pH-unstable NA. This result is the same as the results for viruses with low-pH-stable NA including H3N2 virus, H2N2 virus [8], and H1N1 virus [7]. We previously investigated which step(s) of the infection process was associated with the low-pH stability in NA of 1968 H3N2 virus and 1968 H2N2 virus. The low-pH stability of their NAs significantly affected virus yield/cell [8]. For pandemic (H1N1) 2009 virus, the low-pH stability of NA is thought to be due to enhancement of virus replication in cells.

We have investigated replicabilities of reverse genetics viruses of WSN strain backbone possessing low-pH-stable N2 NA or low-pH-unstable N2 NA in a mouse model. WSN strain is known to have high level of virulence and lethality in mice. In the lungs of infected mice, the viruses possessing low-pH-stable NA showed much higher replicability than did the viruses possessing low-pH-unstable NA. These viruses were not detected in organs other than the lungs in mice [8]. Thus, our previous study has already indicated that enhancement of virus replication by low-pH-stable NA is not limited to MDCK cells. In this paper, we checked replicability of reverse genetics viruses of pandemic (H1N1) 2009 strain backbone possessing low-pH-stable NA or low-pH-unstable NA in human lung adenocarcinoma Calu-3 cells. As was found in MDCK cells, the virus possessing low-pH-stable NA also showed high replicability in Calu-3 cells compared to the virus possessing low-pH-unstable NA. This result also indicates that enhancement of virus replication by low-pH-stable NA is not limited to MDCK cells.

In our previous studies, the NAs of all pandemic human viruses in 1918, 1957, and 1968 were low-pH-stable [4]-[8]. The low-pH-stable NA of pandemic viruses 1918 H1N1 and 1968 H3N2 enhanced replication of reverse genetics viruses with a backbone of WSN strain in cells and in a mouse model [7], [8]. Phylogenetic analysis and investigation of the low-pH stabilities of NA indicated that the low-pH-stable NA of pandemic 1968 H3N2 virus was inherited from the low-pH-stable NA of H2N2 virus and replaced with the low-pH-unstable NA of H2N2 virus until 1971 [6]. Since that investigation was carried out only on a year scale, the detailed relationship between low-pH-stable NA and a pandemic remained unknown. In the present study, the NAs of some pandemic 2009 (H1N1) viruses were more low-pH stable than were the NAs of seasonal H1N1 viruses. Our previous studies found that all of three pandemic viruses (1918 H1N1, 1957 H2N2, and 1968 H3N2) in the 20^th^ century had low-pH-stable NA. In this paper, low-pH-stable NA was also confirmed in 2009 pandemic virus. It was also confirmed that low-pH-stable NA enhanced replication of reverse genetics virus even with a backbone of pandemic 2009 (H1N1) virus. For this pandemic, we can investigate transition of the low-pH stability of NA not only on a year scale but also on a day or month scale at an early stage of the pandemic. Determination of NA mutations that induced low-pH stability enabled phylogenic analysis with predictive transition of the low-pH stability from 2009-2010 H1N1 NA genes in a databank. At an early stage of the 2009 pandemic in the United States, the NAs were low-pH unstable until early April in 2009, but almost all of the isolates had low-pH-stable NA caused by two mutations from late April in 2009. At an early stage of a pandemic, acquisition of low-pH-stable NA in a pandemic candidate virus might be one of factors promoting the pandemic at an early stage of the pandemic. What is the meaning of the transient gain of low-pH stability in NA? Enhancement of virus replication might contribute to spread among humans not immunized against new subtype viruses, through low-pH-stable NA that can be acquired from only one or a few mutations in NA. Once new subtype viruses have spread worldwide, the viruses would be difficult to maintain among many humans immunized against the viruses. The viruses might require a decrease of virus replicability to maintain constant antigenic variations and infections under the condition of many immunized humans (epidemic), through low-pH-unstable NA. The present study provides new insights into the mechanism underlying the occurrence of a pandemic.

## Supporting Information

Table S1 **Accession numbers and Amino acid residues at positions 106 and 248 in NA genes of pandemic (H1N1) 2009 viruses and swine H1N1 influenza A viruses used to generate phylogenic tree.**
(DOC)

Table S2 **Accession numbers and Amino acid residues at positions 106 and 248 in NA proteins of 90 human H1N1 viruses isolated from September 2010 to March 2011.**
(DOC)
