# Supplementary material for: Mutations in NA That Induced Low pH-Stability and Enhanced the Replication of Pandemic (H1N1) 2009 Influenza A Virus at an Early Stage of the Pandemic
Source: PLoS One. 2013 May 16;8(5):e64439. doi: 10.1371/journal.pone.0064439 (PMC3655982; doi:10.1371/journal.pone.0064439)
Supplement: Table S1 — Accession numbers and Amino acid residues at positions 106 and 248 in NA genes of pandemic (H1N1) 2009 viruses and swine H1N1 influenza A viruses used to generate phylogenic tree. (DOC) [file pone.0064439.s001.doc]

Table S1. Accession numbers and Amino acid residues at positions 106 and 248 in NA genes of pandemic (H1N1) 2009 viruses and swine H1N1 influenza A viruses used to generate phylogenic tree.

Strain (H1N1) Accession NA amino acid position Collection

Number 106 248 Date

Swine viruses

A/swine/Italy/247578/2004 EU045393 Val Asn -

A/swine/Italy/65296/2004 EU045389 Val Asn -

A/swine/Spain/53207/2004 CY010582 Val Asn -

A/swine/Hungary/19774/2006 FJ798780 Val Asn -

Pandemic (H1N1) 2009 viruses

A/Texas/09/2009 GQ168670 Val Asn 25 Apr.

A/Texas/22/2009 GQ160571 Val Asn 26 Apr.

A/Mexico/4176/2009 GQ162181 Val Asn 13 Apr.

A/California/04/2009 FJ966084 Val Asn 01 Apr.

A/California/09/2009 FJ966973 Val Asn 15 Apr.

A/Texas/15/2009 GQ122096 Val Asn 15 Apr.

A/Mexico/4108/2009 GQ162169 Val Asn 03 Apr.

A/Mexico/4115/2009 GQ149691 Val Asn 07 Apr.

A/Texas/04/2009 GQ377071 Val Asn 14 Apr.

A/Texas/05/2009 GQ457486 Val Asn 15 Apr.

A/Mexico/3955/2009 GQ162193 Val Asn 02 Apr.

A/Mexico/InDRE4487/2009 FJ998214 Val Asn 14 Apr.

A/Mexico/4269/2009 GQ162171 Val Asn 15 Apr.

A/Texas/06/2009 FJ984383 Val Asn 23 Apr.

A/Texas/08/2009 GQ168632 Val Asn 24 Apr.

A/South Carolina/09/2009 GQ221795 Val Asn 26 Apr.

A/Arizona/01/2009 GQ117064 Val Asn 22 Apr.

A/New York/1682/2009 CY039903 Val Asn 27 Apr.

A/California/06/2009 FJ971075 Val Asn 16 Apr.

A/California/05/2009 FJ966956 Val Asn 30 Mar.

A/Nebraska/02/2009 GQ221802 Val Asn Apr.

A/Colorado/03/2009 GQ221813 Val Asn 27 Apr.

A/Mexico/InDRE4114/2009 GQ132155 Val Asn -

A/Arizona/02/2009 GQ221789 Val Asn 26 Apr.

A/Minnesota/02/2009 GQ117071 Val Asn 27 Apr.

A/Netherlands/602/2009 CY039528 Val Asn 29 Apr.

A/Mexico/4486/2009 GQ162201 Val Asn 14 Apr.

A/Wisconsin/WSLH26327/2009 Current study Val Asn -

A/Indiana/09/2009 GQ117094 Val Asn 22 Apr.

A/Korea/01/2009 GQ132185 Ile Asn 02 May

A/Osaka/180/2009 GQ365445 Ile Asn -

A/Omsk/01/2009 GQ527169 Ile Asn -

A/Almati/01/2009 GQ485663 Ile Asn -

A/Amagasaki/2/2009 GQ220729 Ile Asn -

A/Hyogo/1/2009 GQ220731 Ile Asn 17 May

A/Kobe/1/2009 GQ220733 Ile Asn -

A/Nanjing/1/2009 GQ504753 Ile Asn 18 Jun.

A/Niigata/700/2009 GU014765 Ile Asn 22 Jun.

A/Saitama/43/2009 AB514228 Ile Asn 21 May

A/Guangdong/06/2009 HM780472 Val Asp 29 May

A/Lyon/48.49/2009 JF429400 Val Asp 23 Nov.

A/Pensacola/INS213/2009 CY066777 Val Asp 17 Nov.

A/Lebanon/09L-28/2009 AB603640 Val Asp 03 Nov.

A/New York/18/2009 FJ984350 Ile Asp 25 Apr.

A/New York/12/2009 GQ168672 Ile Asp 25 Apr.

A/Ohio/07/2009 GQ323567 Ile Asp 24 Apr.

A/New York/11/2009 GQ168651 Ile Asp 25 Apr.

A/Massachusetts/06/2009 GQ117042 Ile Asp 26 Apr.

A/New York/20/2009 GQ117084 Ile Asp 25 Apr.

A/New York/31/2009 GQ168660 Ile Asp 24 Apr.

A/New York/19/2009 FJ984390 Ile Asp 25 Apr.

A/New York/15/2009 GQ457483 Ile Asp 25 Apr.

A/New York/10/2009 FJ984371 Ile Asp 25 Apr.

A/Massachusetts/07/2009 GQ117102 Ile Asp 25 Apr.

A/New York/22/2009 GQ117022 Ile Asp 24 Apr.

A/Mexico/4575/2009 GQ162196 Ile Asp 20 Apr.

A/New York/06/2009 FJ984340 Ile Asp 25 Apr.

A/California/14/2009 GQ117036 Ile Asp 25 Apr.

A/Texas/23/2009 GQ160547 Ile Asp 27 Apr.

A/New York/23/2009 GQ168627 Ile Asp 24 Apr.

A/Mexico/4593/2009 GQ162173 Ile Asp 20 Apr.

A/Michigan/02/2009 GQ117108 Ile Asp 26 Apr.

A/Mexico/4604/2009 GQ149631 Ile Asp 19 Apr.

A/Norway/3568/2009 Current study Ile Asp -

A/Norway/3858/2009 Current study Ile Asp -

Human H1N1 viruses from September 2010 to March 2011

A/Thailand/CU-C1157/2010 CY081157 Ile Asp 8 Sep.

A/Ontario/130741/2010 CY081063 Ile Asp 13 Oct.

A/Moscow/IIV-33/2010 HQ834748 Ile Asp 13 Oct.

A/Thailand/CU-H2698/2010 CY089457 Ile Asp 3 Nov.

A/Ontario/3620/2010 CY081071 Ile Asp 24 Nov.

A/Vladivistok/7/2010 HQ891282 Ile Asp 3 Dec.

A/Shahriar/5336/2010 HQ712176 Ile Asp 6 Dec.

A/Ghom/169/2010 JF500427 Ile Asp 29 Dec.

A/Ulaanbaatar/190/2011 CY080574 Ile Asp 11 Jan.

A/Beijing/HZ01/2011 JF316715 Ile Asp 14 Jan.

A/Murmansk/RII1/2011 CY091617 Ile Asp 26 Jan.

A/Voronezh/RII1/2011 CY091606 Ile Asp 9 Feb.

A/Singapore/TT134/2011 CY091713 Ile Asp 14 Feb.

A/Singapore/GP730/2011 CY091701 Ile Asp 2 Mar.

A/Mexico/InDRE1946/2011 CY089390 Ile Asp 12 Mar.

-, unkown.
