# Supplementary material for: Mutations in NA That Induced Low pH-Stability and Enhanced the Replication of Pandemic (H1N1) 2009 Influenza A Virus at an Early Stage of the Pandemic
Source: PLoS One. 2013 May 16;8(5):e64439. doi: 10.1371/journal.pone.0064439 (PMC3655982; doi:10.1371/journal.pone.0064439)
Supplement: Table S2 — Accession numbers and Amino acid residues at positions 106 and 248 in NA proteins of 90 human H1N1 viruses isolated from September 2010 to March 2011. (DOC) [file pone.0064439.s002.doc]

Table S2. Accession numbers and Amino acid residues at positions 106 and 248 in NA proteins of 90 human H1N1 viruses isolated from September 2010 to March 2011.

Strain (H1N1) Accession NA amino acid position Collection

Number 106 248 Date

A/Beijing/3848/2010 ADW95306 Ile Asp Nov.

A/Beijing/3884/2010 ADW95327 Ile Asp Nov.

A/Beijing/3907/2010 ADW95333 Ile Asp -

A/Beijing/HZ01/2011 ADX87399 Ile Asp 14 Jan.

A/Czech Republic/1/2011 ADZ75318 Ile Asp 25 Jan.

A/Denmark/106/2010 ADV69050 Ile Asp 4 Dec.

A/Denmark/110/2010 ADV69051 Ile Asp 10 Dec.

A/Denmark/118/2010 ADZ52933 Ile Asp 22 Dec.

A/Denmark/120/2010 ADZ52932 Ile Asp 20 Dec.

A/Denmark/123/2010 ADW01474 Ile Asp 23 Dec.

A/Denmark/125/2010 ADW01473 Ile Asp 21 Dec.

A/Denmark/126/2010 ADW01470 Ile Asp 21 Dec.

A/Denmark/129/2010 ADW01472 Ile Asp 26 Dec.

A/Denmark/130/2010 ADW01475 Ile Asp 21 Dec.

A/Denmark/131/2010 ADW01471 Ile Asp 27 Dec.

A/Denmark/132/2010 ADW01476 Ile Asp 27 Dec.

A/Denmark/137/2010 ADW01469 Ile Asp 26 Dec.

A/Denmark/20/2011 ADZ52931 Ile Asp 4 Jan.

A/Denmark/24/2011 ADZ52934 Ile Asp 15 Jan.

A/Denmark/26/2011 ADZ52937 Ile Asp 13 Jan.

A/Denmark/27/2011 ADZ52935 Ile Asp 13 Jan.

A/Denmark/28/2011 ADZ52936 Ile Asp 20 Jan.

A/Denmark/36/2011 AEF58542 Ile Asp 1 Feb.

A/Denmark/72/2011 AEF58512 Ile Asp 7 Feb.

A/Denmark/73/2011 AEF58532 Ile Asp 29 Jan.

A/Ghom/167/2010 ADZ31480 Ile Asp 30 Dec.

A/Ghom/169/2010 ADZ31481 Ile Asp 29 Dec.

A/Ghom/198/2010 ADZ31482 Ile Asp 29 Dec.

A/Ghom/215/2010 ADZ31483 Ile Asp 30 Dec.

A/Kaliningrad/RII4/2011 AEG79836 Ile Asp 27 Jan.

A/Karaj/5327/2010 ADT91184 Ile Asp 6 Dec.

A/Karaj/5607/2010 ADZ31484 Ile Asp 19 Dec.

A/Karaj/5660/2010 ADV19293 Ile Asp 13 Dec.

A/Karaj/5685/2010 ADV19296 Ile Asp 21 Dec.

A/Karaj/5718/2010 ADV19299 Ile Asp 14 Dec.

A/Karaj/6072/2010 ADZ31492 Ile Asp 29 Dec.

A/Mexico/InDRE1946/2011 AEA74034 Ile Asp 22 Mar.

A/Moscow/IIV-33/2010 ADU56206 Ile Asp 13 Oct.

A/Murmansk/RII1/2011 AEG79830 Ile Asp 26 Jan.

A/Ontario/130741/2010 ADW54522 Ile Asp 13 Oct.

A/Ontario/3620/2010 ADW54473 Ile Asp 24 Nov.

A/Ontario/720545/2010 ADW54483 Ile Asp 17 Nov.

A/Ontario/741328/2010 ADW54494 Ile Asp 23 Nov.

A/Ontario/804635/2010 ADW54514 Ile Asp 14 Dec.

A/Saint-Petersburg/RII109/2011 AEG79820 Ile Asp 14 Feb.

A/Saint-Petersburg/RII166/2011 AEG79826 Ile Asp 21 Feb.

A/Saint-Petersburg/RII174/2011 AEG79802 Ile Asp 05 Mar.

A/Saint-Petersburg/RII45/2011 AEG79808 Ile Asp 07 Feb.

A/Shahriar/5336/2010 ADT91186 Ile Asp 06 Dec.

A/Singapore/GP39/2011 AEH59378 Ile Asp 7 Jan.

A/Singapore/GP433/2011 AEH59380 Ile Asp 2 Feb.

A/Singapore/GP4344/2010 AEH59356 Ile Asp 20 Oct.

A/Singapore/GP4444/2010 AEH59358 Ile Asp 9 Nov.

A/Singapore/GP4511/2010 AEH59360 Ile Asp 30 Nov.

A/Singapore/GP4565/2010 AEH59362 Ile Asp 10 Dec.

A/Singapore/GP4610/2010 AEH59366 Ile Asp 20 Dec.

A/Singapore/GP4670/2010 AEH59368 Ile Asp 30 Dec.

A/Singapore/GP4682/2010 AEH59370 Ile Asp 31 Dec.

A/Singapore/GP511/2011 AEH59384 Ile Asp 11 Feb.

A/Singapore/GP536/2011 AEH59386 Ile Asp 14 Feb.

A/Singapore/GP582/2011 AEH59388 Ile Asp 16 Feb.

A/Singapore/GP62/2011 AEH59390 Ile Asp 10 Jan.

A/Singapore/GP730/2011 AEH59392 Ile Asp 2 Mar.

A/Singapore/KK105/2011 AEH59394 Ile Asp 11 Feb.

A/Singapore/KK124/2011 AEH59398 Ile Asp 22 Feb.

A/Singapore/KK68/2011 AEH59400 Ile Asp 25 Jan.

A/Singapore/KK734/2010 AEH59372 Ile Asp 17 Dec.

A/Singapore/SGH01/2011 AEH59402 Ile Asp 2 Feb.

A/Singapore/TT134/2011 AEH59404 Ile Asp 14 Feb.

A/Singapore/TT142/2011 AEH59406 Ile Asp 21 Feb.

A/Singapore/TT160/2011 AEH59408 Ile Asp 25 Feb.

A/Singapore/TT164/2011 AEH59410 Ile Asp 3 Mar.

A/Singapore/TT496/2010 AEH59374 Ile Asp 20 Dec.

A/Singapore/TT5/2011 AEH59412 Ile Asp 3 Jan.

A/Singapore/TT67/2011 AEH59414 Ile Asp 21 Jan.

A/Singapore/TT89/2011 AEH59416 Ile Asp 25 Jan.

A/Switzerland/5165/2010 ADU25480 Val Asn 21 Oct.

A/Tehran/5675/2010 ADV19295 Ile Asp 18 Dec.

A/Tehran/5958/2010 ADZ31488 Ile Asp 27 Dec.

A/Thailand/CU-C1157/2010 ADW65826 Ile Asp 7 Sep.

A/Thailand/CU-H2358/2010 ADX20814 Ile Asp 8 Sep.

A/Thailand/CU-H2389/2010 ADX20815 Ile Asp 10 Sep.

A/Thailand/CU-H2417/2010 AEA72779 Ile Asp 13 Sep.

A/Thailand/CU-H2543/2010 AEA72789 Ile Asp 25 Sep.

A/Thailand/CU-H2548/2010 AEB21340 Ile Asp 27 Sep.

A/Thailand/CU-H2698/2010 AEB21350 Ile Asp 3 Nov.

A/Thailand/CU-H2911/2011 AEB21360 Ile Asp 20 Jan.

A/Ulaanbaatar/190/2011 ADV78132 Ile Asp 11 Jan.

A/Vladivistok/7/2010 ADV69068 Ile Asp 3 Dec.

A/Voronezh/RII1/2011 AEG79814 Ile Asp 9 Feb.
